# Electrical resistivity imaging data for hydrogeological and geological investigations of Szuszalewo peatland (North-East Poland)

**DOI:** 10.1016/j.dib.2024.110626

**Published:** 2024-06-11

**Authors:** Łukasz Kaczmarek, Grzegorz Sinicyn, Krzysztof Kochanek, Bartosz Bednarz, Mateusz Grygoruk, Maria Grodzka-Łukaszewska

**Affiliations:** a Faculty of Building Services, Hydro and Environmental Engineering, Warsaw University of Technology, Warsaw, Poland; b Institute of Geophysics, Polish Academy of Sciences, Warsaw, Poland; c Institute of Environmental Engineering, Warsaw University of Life Sciences, Warsaw, Poland

**Keywords:** Geophysics, Peat, Water flow, River valley

## Abstract

This publication contains data on geophysical measurements taken in the Szuszalewo wetlands located in northern Poland. The measurements were made using the electrical resistivity imaging (ERI) method. The ERI data was collected during two survey expeditions – March 30th, 31st (two ERI profiles), April 1st (one ERI profile), and May 12th (two prospection lines) 2023. The reason goal was to illustrate the arrangement of geological layers creating this wetland. The data repository contains detailed data descriptions for each survey site. This Electrical Resistivity Imaging (ERI) data from the selected survey sites can be used to perform numerical modeling of groundwater and surface water interaction in this environmentally valuable area, which is, to a certain extent a scientific terra incognita, hydrogeological investigation of hydraulic conductivity and hydrodynamic field, identify geological structure, and characterize engineering properties of the organic soils.

Specifications Table

**Table:** 

Subject	Geophysics
Specific subject area	Electrical resistivity imaging
Data format	Raw, Inverted model
Type of data	Table, Image, Graph, Figure
Data collection	The resistivity imaging data for the wetlands Szuszalewo were collected during two survey expeditions – March 30th, 31st (two ERI profile), April 1st (one ERI profile) and May 12th (two prospection lines) 2023. The goal of the second expedition was to verify part of the affected results (due to weather conditions) and refine the variability of the geological structure. The data repository contains detailed data descriptions for each survey site.
Data source location	Faculty of Building Services, Hydro and Environmental Engineering, Warsaw University of Technology, Warsaw, Poland
Data accessibility	Repository name: Mendeley data Data identification number: 10.17632/363vtnz5np.1 Direct URL to data: https://data.mendeley.com/datasets/363vtnz5np/1

## 1 Value of the Data

• The Electrical Resistivity Imaging (ERI) data from the selected survey sites can be used to perform numerical modeling of groundwater and surface water interaction (i.e. [1,2]) in this environmentally valuable area which is to a certain extend a scientific terra incognita, hydrogeological investigations of hydraulic conductivity and hydrodynamic field, identify geological structure, and characterize engineering properties of the organic soils.
• The ERI data can be used to monitor groundwater heads [3] (whether generally hydrogeological conditions; i.e. [4]) as well as terrain changes (subsidence) by comparing with future survey findings. It can be related to the climate change effects.
• The ERI data can be jointly inverted and interpreted with different field measurement (i.a. other geophysical) data to obtain more reliable subsurface information. Studies like recognition drilling and probing (i.e. [5]), sampling and hydraulic conductivity lab tests [6], low-flow filed pumping tests, seismic, electromagnetic, and ground penetrating radar can be effectively integrated with ERI data
• By means of open-source inversion algorithms, can raw ERI data be reprocessed to generate 2D and 3D inverted models (i.e. [7]). Machine learning and statistical algorithms can be used to further interpretation of the inverted resistivity data.

## 2 Background

Peatlands constitute unique areas of the interaction between groundwater and surface water. The environmentally extremely valuable organic soils, due to their accumulation properties (water, carbon dioxide, organic matter), high compressibility and water permeability depending on the tension level, constitute a great challenge in planning their protection and possible development. Due to challenging availability and high variability of geological conditions, all the data considering wetlands become a very important input for further analyses, numerical calculations, field and laboratory tests. The study area where we attempted to recognize the geological structure of mess peat and sedge peat with reed includes also an educational path in the Biebrza National Park near the Szuszalewo village (N-E Poland). Mostly because of the environmental uniqueness of this case study area, the non-invasive method of electrical resistivity tomography (which does not need heavy transportation to be moved), fits perfectly into the circumstances of achievable tests (i.e. [8]). The research is of reference nature for peat bogs occurring in this part of Europe, because of the scope of research and the research techniques used which additionally allowed for continuous identification of soil variability in the subsoil area. The peatland developed at the foot of moraine elevations (north and south) of the Biebrza River bounding the peatland from the east and west. Rural buildings and an motorway are located in the vicinity the wetlands, representing the most likely sources of anthropopression. All these make this area representative for many similar peatlands in northeastern Poland, one of the largest intact peatlands in Europe.

## 3 Data Description

**Fig. 1:**
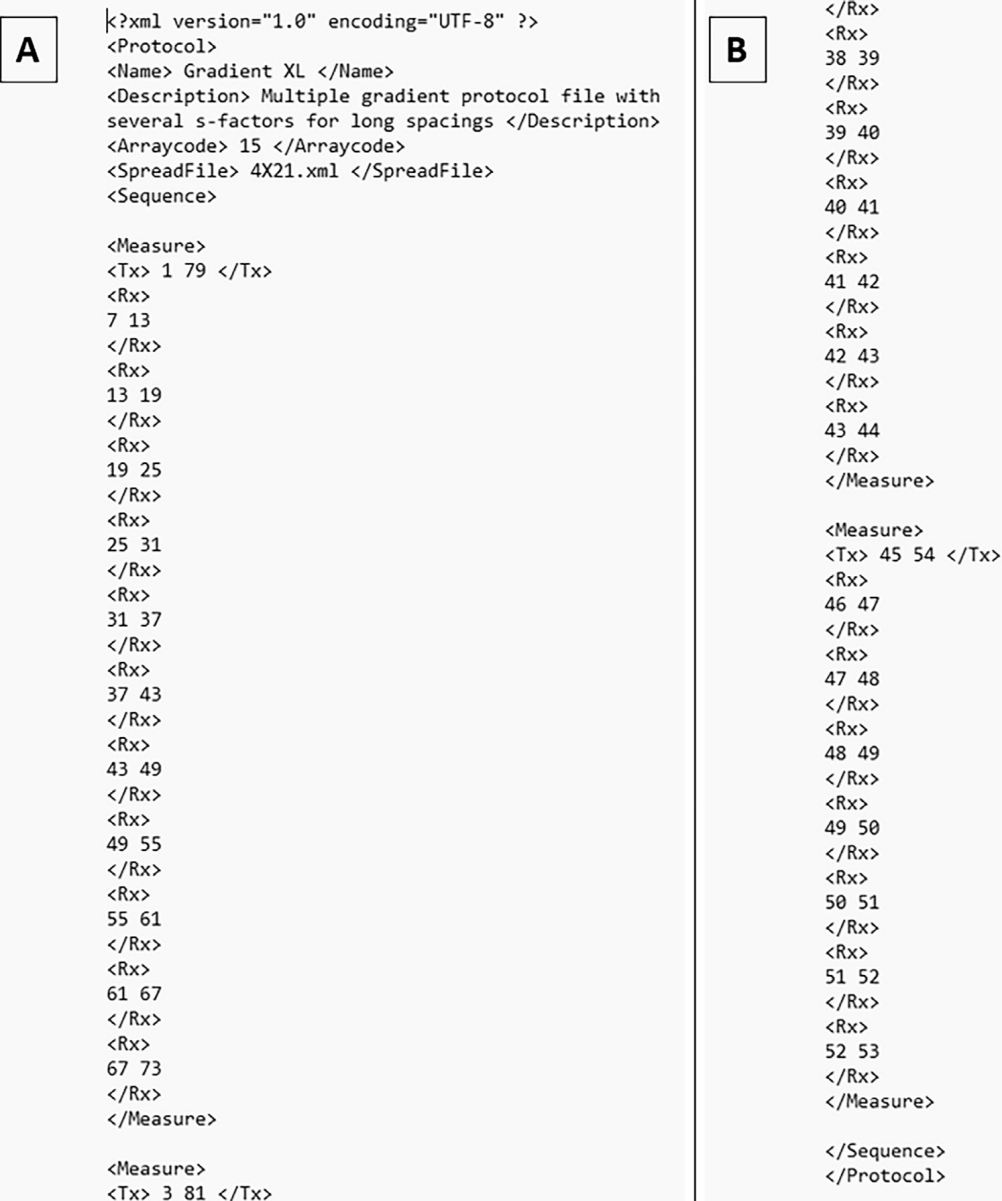
The used Gradient_XL protocol with several stations as a roll-along: begging (A) and end (B) of the protocol.

The data consider electrical resistivity imaging results for hydrogeological and geological investigations conducted in reference Polish location. They can be easily accessible at Mendeley's data repository [9]: https://data.mendeley.com/datasets/363vtnz5np/1. The repository is divided into five major folders: ERI Data: 1 Prospection Line A, 2 Prospection Line B, 3 Prospection Line C, 4 Prospection Line D and 5 Prospection Line E. The ERI Data Folders include raw data (presented general array format; in "dat" files) and image of the inverted resistivity models for each ERI profile (presented in “jpg” files; with default colour scale - linear/logarithmic contour intervals of the processing last iteration). The Google Earth KML files with the location of the ERI profiles are also provided for each survey site. The all the ERI profiles have been measured by the same ABEM Terrameter LS-2 setup by Multiple gradient array (Roll-along technique used in case of all profiles; “Gradient_XL” protocol with several stations – Fig. 1) with 2 m electrode spacing.

**Fig. 2:**
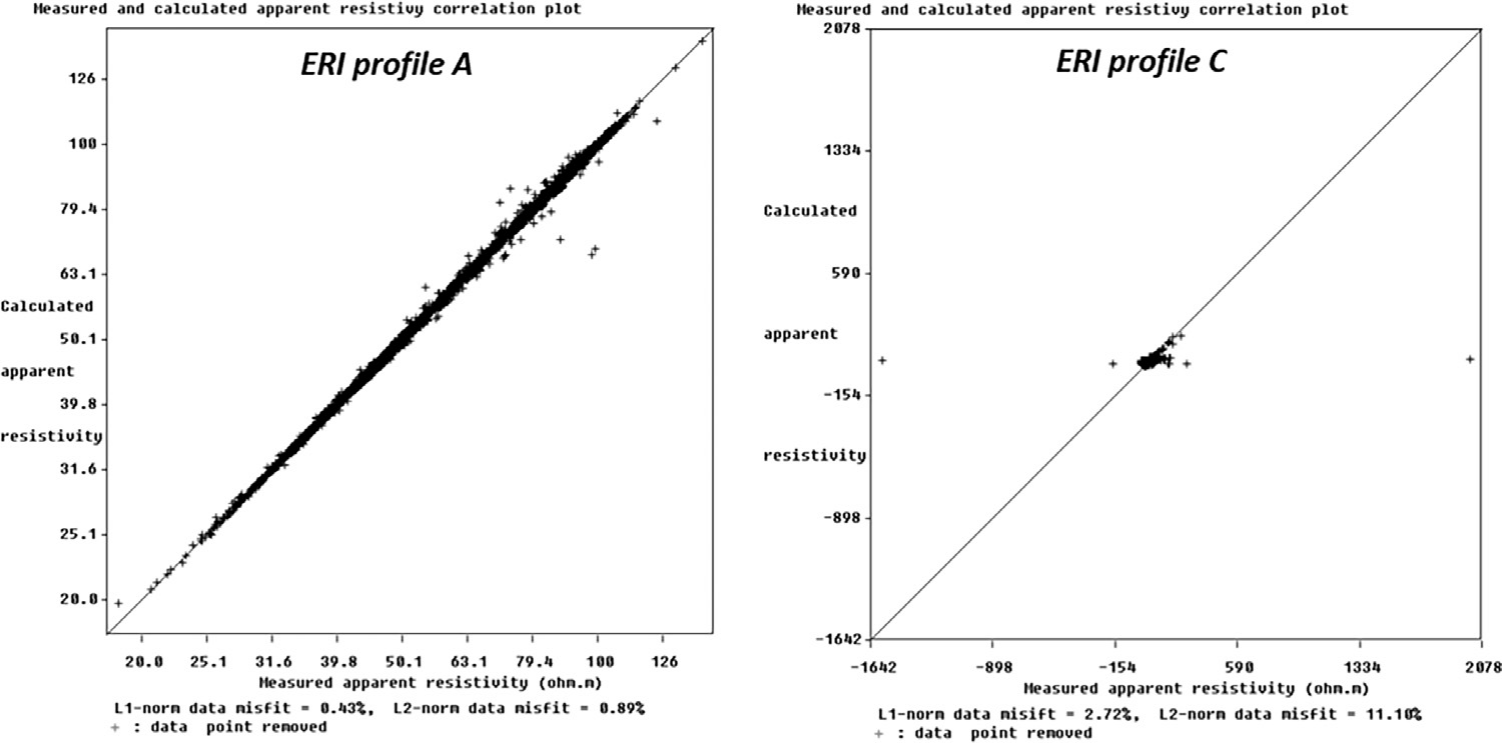
The measured and calculated apparent resistivity correlation plot of the data register in the case of ERI profiles A and C.

The data from profiles A, D, and E is good quality because the misfit between measured and predicted resistivity data reached RMS (the root mean square) error (information about the difference between the measured and calculated apparent resistivity values) values less than 1%. In the case of profiles B and C, the quality will force some data filters (like removing registered data with too high variance, too low electrical voltage, and “obviously too-high or too-low apparent resistivity values). In this case, we had some difficulties with weather (periods of light rainfall - longer in the case of profile C). The RMS error values are less than 16%. Fig. 2 shows an exemplary measured and calculated apparent resistivity correlation plot of the data register in the field (generated by Res2Dinv without the aforementioned date filter).

**Fig. 3:**
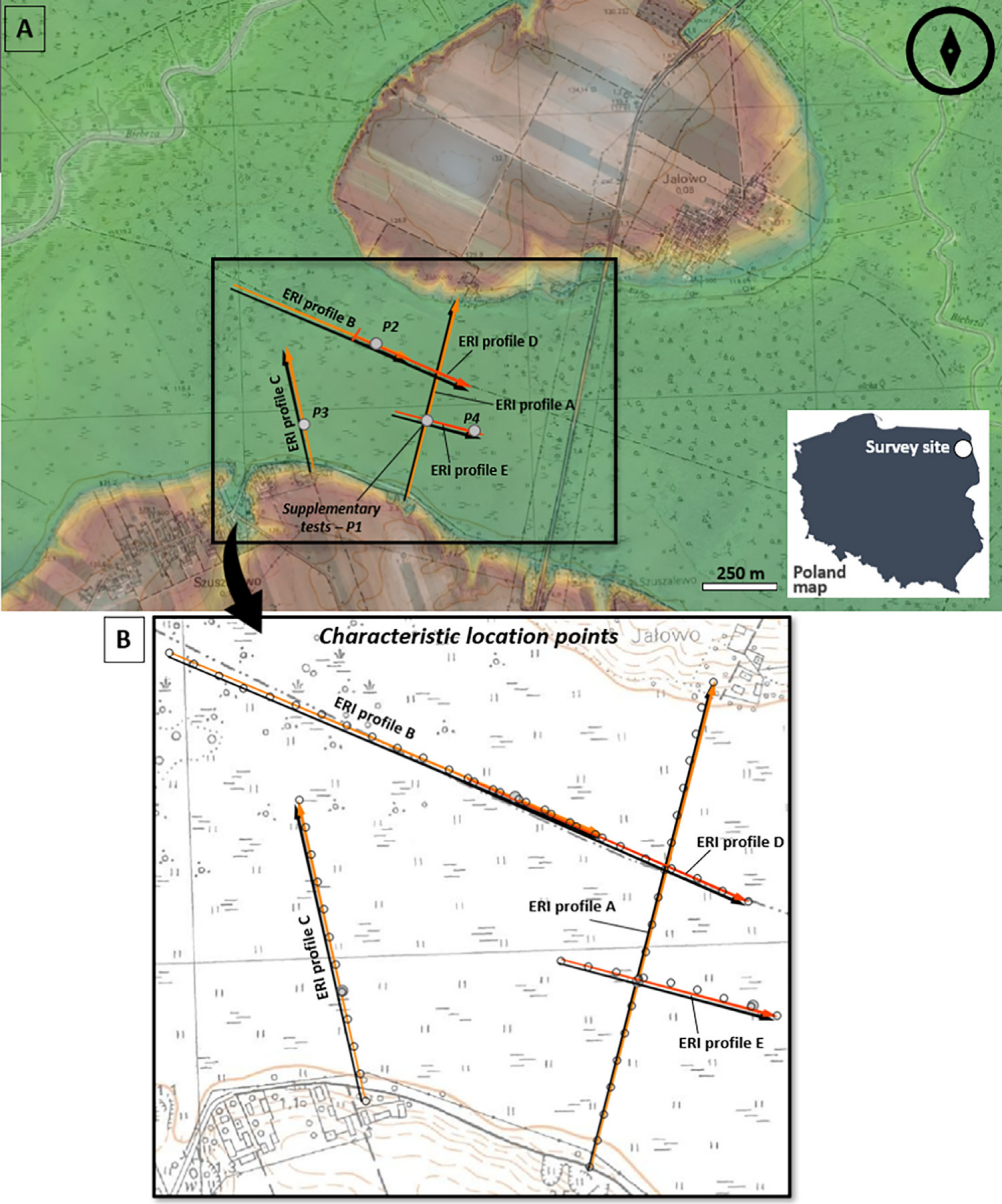
The ERI data distribution for hydrogeological and geological studies and in the background topographic map (source: www.geoportal.gov.pl): A - broader scope of the research area (where green colour means the lowest elevation, where white the highest one), B - ERI profiles with GPS measurement characteristics points.

Below one can find the Table 1, Table 2, Table 3 field photos, reference resistivity table, location map (Fig. 3) and selected exemplary results. Table 1 presents details on the ERI's metadata: profile line name, survey data, profile orientation, array type, electrode spacing, profile length, file name and type, instrument info. Table 2 shows the coordinates of each characteristic point of the ERI profiles (beginning and ending, all Terrameter station positions which are equivalent to the earlier or later presence of a connector). The name of each point contains “Sz” from location *Szuszalewo*, next the letter indicates specific ERI profile and in the end the number of measurement point (from beginning to the end of ERI prospection line). Additionally, Table 3 contain details on supplementary tests: shallow recognition drillings (location coordinates, depth, soil profile, water table depth), basic surface and near surface water parameters (pH, temperature, electrical conductivity of water).

**Table 1:** ERI data resume descriptions for the Szuszalewo site.

Profile line name	Survey date (dd/mm/year)	Profile orientation	Profile length (m)	File name (and type)
A	30/03/2023	SW->NE	720	ERI profile A (dat)
B	31/03/2023	NW->SE	680	ERI profile B (dat)
C	01/04/2023	SE->NW	440	ERI profile C (dat)
D	12/05/2023	NW->SE	440	ERI profile D (dat)
E	12/05/2023	NW->SE	320	ERI profile E (dat)

**Table 2:** The coordinates of ERI prospection lines characteristic points.

Name	ERI profile coordinates (EPSG 2180)		Elevation m a.s.l. (PL-KRON86-NH)
	Latitude (N)	Longitude (E)	
SZ-A-0	658,569.90	787,433.64	120.90
SZ-A-1	658,608.74	787,442.92	119.94
SZ-A-2	658,646.80	787,452.35	119.78
SZ-A-3	658,685.37	787,461.88	119.57
SZ-A-4	658,724.30	787,471.57	119.61
SZ-A-5	658,763.66	787,482.14	119.53
SZ-A-6	658,802.86	787,492.30	119.51
SZ-A-7	658,841.89	787,502.35	119.62
SZ-A-8	658,880.79	787,512.67	119.62
SZ-A-9	658,919.55	787,521.88	119.55
SZ-A-10	658,959.52	787,530.62	119.55
SZ-A-11	658,998.76	787,539.60	119.60
SZ-A-12	659,038.27	787,549.52	119.51
SZ-A-13	659,076.91	787,557.90	119.47
SZ-A-14	659,116.22	787,566.83	119.49
SZ-A-15	659,155.41	787,575.97	119.46
SZ-A-16	659,194.37	787,585.29	119.50
SZ-A-17	659,232.91	787,592.98	119.61
SZ-A-18	659,267.55	787,611.65	121.81
SZ-B-0	659,310.92	786,828.23	119.33
SZ-B-1	659,295.25	786,861.12	119.36
SZ-B-2	659,278.11	786,897.31	119.32
SZ-B-3	659,259.54	786,932.44	119.44
SZ-B-4	659,247.87	786,970.46	119.41
SZ-B-5	659,235.95	787,008.19	119.44
SZ-B-6	659,222.90	787,044.95	119.41
SZ-B-7	659,205.98	787,081.40	119.51
SZ-B-8	659,189.82	787,117.72	119.57
SZ-B-9	659,174.51	787,154.72	119.40
SZ-B-10	659,159.80	787,191.78	119.42
SZ-B-11	659,142.67	787,228.52	119.43
SZ-B-12	659,126.10	787,265.53	119.48
SZ-B-13	659,110.60	787,302.37	119.46
SZ-B-14	659,096.66	787,339.79	119.51
SZ-B-15	659,079.40	787,376.39	119.46
SZ-B-16	659,061.24	787,412.44	119.45
SZ-B-17	659,047.03	787,454.94	119.49
SZ-C-0	658,669.82	787,107.29	122.72
SZ-C-1	658,704.82	787,100.58	119.69
SZ-C-2	658,743.98	787,091.43	119.57
SZ-C-3	658,783.79	787,082.86	119.56
SZ-C-4	658,822.97	787,074.58	119.56
SZ-C-5	658,862.43	787,065.37	119.54
SZ-C-6	658,901.99	787,056.64	119.55
SZ-C-7	658,941.58	787,048.39	119.55
SZ-C-8	658,981.14	787,039.18	119.57
SZ-C-9	659,020.96	787,030.50	119.60
SZ-C-10	659,060.37	787,021.76	119.55
SZ-C-11	659,099.17	787,011.09	119.39
SZ-D-0	659,129.40	787,255.58	119.52
SZ-D-1	659,114.44	787,292.57	119.45
SZ-D-2	659,100.56	787,330.19	119.48
SZ-D-3	659,085.60	787,367.35	119.46
SZ-D-4	659,066.64	787,403.39	119.49
SZ-D-5	659,049.99	787,439.79	119.49
SZ-D-6	659,033.25	787,476.54	119.50
SZ-D-7	659,014.23	787,512.22	119.46
SZ-D-8	659,000.42	787,549.75	119.24
SZ-D-9	658,986.52	787,587.29	119.44
SZ-D-10	658,968.26	787,623.27	119.40
SZ-D-11	658,953.56	787,658.92	119.44
SZ-E-0	658,869.73	787,390.70	119.56
SZ-E-1	658,859.12	787,430.00	119.55
SZ-E-2	658,851.52	787,469.71	119.55
SZ-E-3	658,843.32	787,509.22	119.44
SZ-E-4	658,835.57	787,548.70	119.49
SZ-E-5	658,826.18	787,587.48	119.51
SZ-E-6	658,813.87	787,625.76	119.55
SZ-E-7	658,801.89	787,664.89	119.58
SZ-E-8	658,788.01	787,700.08	119.59

**Table 3:** Results of supplementary tests performed in the Szuszalewo site.

Point name	Survey date (dd/mm/year)	Coordinates (X;Y in EPSG 2180)	Elevation (m a.s.l. in PL-EVRF2007-NH)	Related ERI profile	Results of tests
P1	30/03/2023	658,838.39; 787,500.91	119.74	A, E	Drilling up to 2 m b.t.s. (peat)
P2	12/05/2023	659,102.44; 787,324.19	119.80	B, D	Drilling up to 2 m b.t.s. (peat); Surface water parameters: T=12.4 °C; pH=6.79; EC=187µS/cm (0.02 Ωm); TDS=94 ppm
P3	1/04/2023	658,824.88; 787,075.73	119.75	C	Drilling up to 2 m b.t.s. (peat)
P4	12/05/2023	658,803.05; 787,668.28	119.90	E	Drilling up to 1 m b.t.s. (peat); Surface water parameters: T=10.4 °C; pH=6.85; EC=203µS/cm (0.02 Ωm); TDS=101 ppm

## 4 Experimental Design, Materials and Methods

The two-dimensional resistivity imaging data were collected by galvanically injecting a low-frequency electrical current into the ground via two electrodes and measuring the voltage difference between two potential electrodes (methodology based on [10]). Differences in resistivity values caused by the flow of electric current through various subsurface mediums are used to identify materials (like i.e. materials listed in Tab. 4). Electrical resistivity of the subsurface material is related to the nature of the soil composition (particle size distribution, mineralogy), the structure (porosity, pore size distribution, connectivity), fluid content, concentration of dissolved electrolytes, content of clay, and temperature [[11], [12], [13]]. Table 4 depicts the electrical resistivity (inverse to conductivity) characteristics of common subsurface geological materials in this area of Poland.

**Table 4:** Typical electrical properties of resistivity (inverse of conductivity) of common geological materials and other mediums in Poland (based on [12] and authors experience).

Soil or other medium	Typical range of electroresistivity (Ωm)
Clayey deposits (clay, till with clay)	<25
Organic soil (peat, alluvion)	10–100 (in aeration zone: 30–100; in saturation zone: 10–50)
Tills and loams	25–70
Sandy deposits	70–1000 (in aeration zone: 200–1000; in saturation zone: 90–250)
Surface water	0.1–300
Rainwater	30–1000
Mineralised water (i.a. sea water)	0.1–5
Permafrost	high

**Fig. 4:**
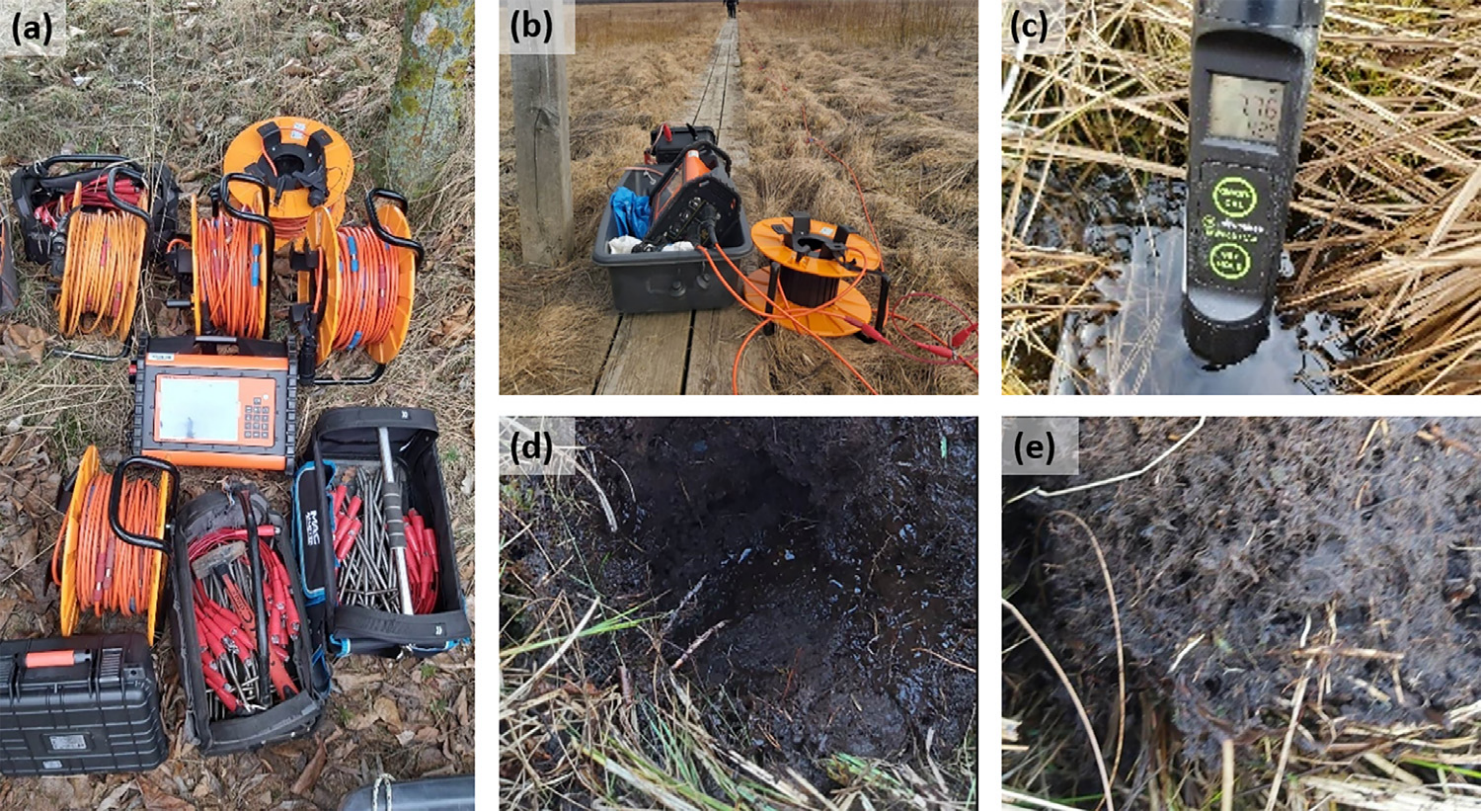
Acquiring ERI field data with multi-electrode (a) 12-point light Abem Terrameter LS-2 set-up, (b) transport of equipment by means of wheelless sleighs; (c) surface water measurements; (d) shallow drilling P1; (e) peat type in P1.

The advanced multi-electrode resistivity sensors were used to measure numerous data points in a single ERI profile by automatica change of the current and potential electrodes. A multiple-gradient array was used to collect resistivity data in the forward and backward survey directions. Fig. 6 depicts the acquisition of field ERI data with the one of the most advanced 12-point light ABEM Terrameter LS-2 setup (Fig. 4a). The electrodes were stuck along the profiles and connected to cables with the cable joints for 21 take-out cables, which lead to a resistivity meter during resistivity measurements. Thus, it is an 81-electrode spread (4×21 take-out cables with a 1 take-out overlap between cables) resulting in a total length of 160 m, when using 2 m spacing. The last take-out from the first cable is in the same place as the first take-out of the second electric cable. The roll-along technique was employed to create longer profiles, with 25% of the spread being moved per roll-along station. Each roll-along appends data to a single database stored on the instrument. The ABEM Terramter LS2 software unit merges all survey sections of one ERI profile during the prospection. After the end of the ERI prospection, the proprietary format (of database) is converted to other formats for inversion; in our case, it was the Res2DInv .dat formats. Therefore, all data from a single profile (made up of multiple roll-along stations) are in a single file for inversion.

The electrodes were tested for the contact resistance before each measurement session, and apparent resistivity was measured. Then, apparent resistivity in many of data points can be measured for a single ERI profile (finally, giving the effect of *quasi* continues section). The multiple-gradient array was described in detail i.a. by [10].

After ERI prospection the supplementary research was performed: shallow drillings for verification of peat presence (Fig. 4d), as well as, surface water measurements for its basic properties (Tab. 4; Fig. 4c).

**Fig. 5:**
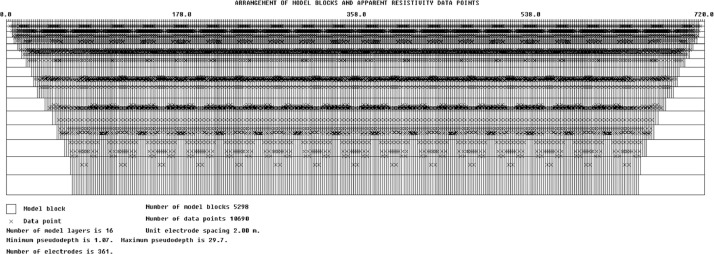
ERI profile A at the Szuszalewo site: the ERI data points distribution.

The measured datasets were optimized: averaging applied to the data based on the positive and negative pulses (in each cycle and between stacks), linear drift correction, and zero-offset were eliminated thanks to the measuring cycle used and fixed before the data was written (during acquisition performed with “maximum number of stacking” parameter equal 2; what was selected cause virgin area with no interference case). The RES2DINV software package [13] was used for data processing and inversion. The smoothness-constrained least-square [14] and robust inversion [15] algorithms were used for data processing, depending on the expected subsurface features, however, the ERI data were mostly inverted by means of the smoothness-constrained least-square inversion algorithm. The iterative inversion method was applied until RMS error dropped below acceptable level (usulay it is 5%), which might be exceeded for surveys in hard rock and noisy environments. As an example, we show the ERI data distribution (Fig. 5) for resistivity data collected from the Szuszalewo peatland (fen) near the Biebrza river – prospection depth: ca. 30 m b.t.s.

**Fig. 6:**
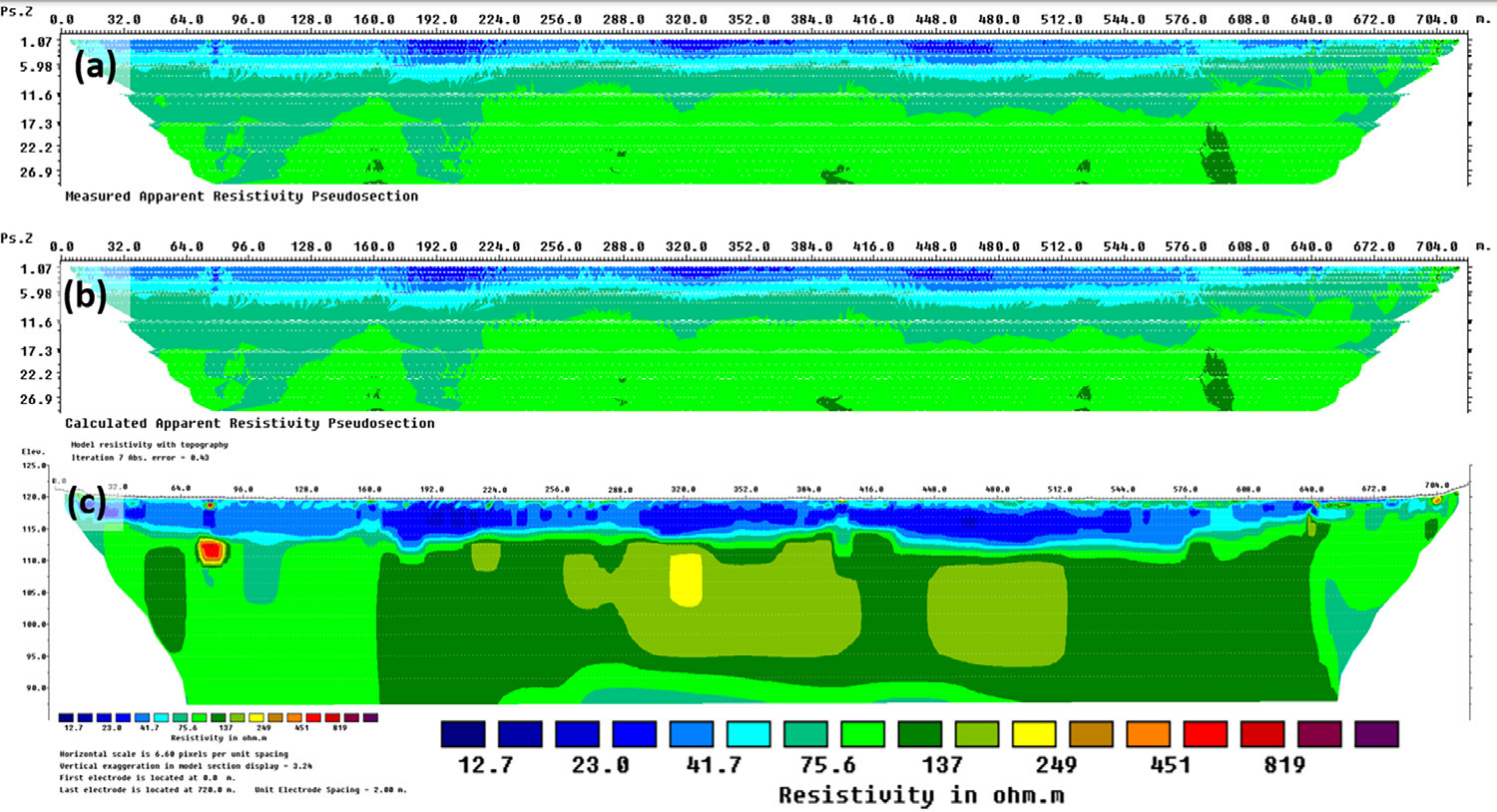
ERI profile A at the Szuszalewo site: (a) apparent resistivity data, (b) calculated apparent resistivity data, and (c) inverted resistivity model.

Using the Roll Along technique, it was possible to obtain very valuable long prospecting lines (several hundred meters long). Fig. 6a shows the distributions of apparent resistivity data, Fig. 6b shows model calculated apparent resistivity data, and Fig. 6c shows an inverted resistivity model.

## Limitations

**Fig. 7:**
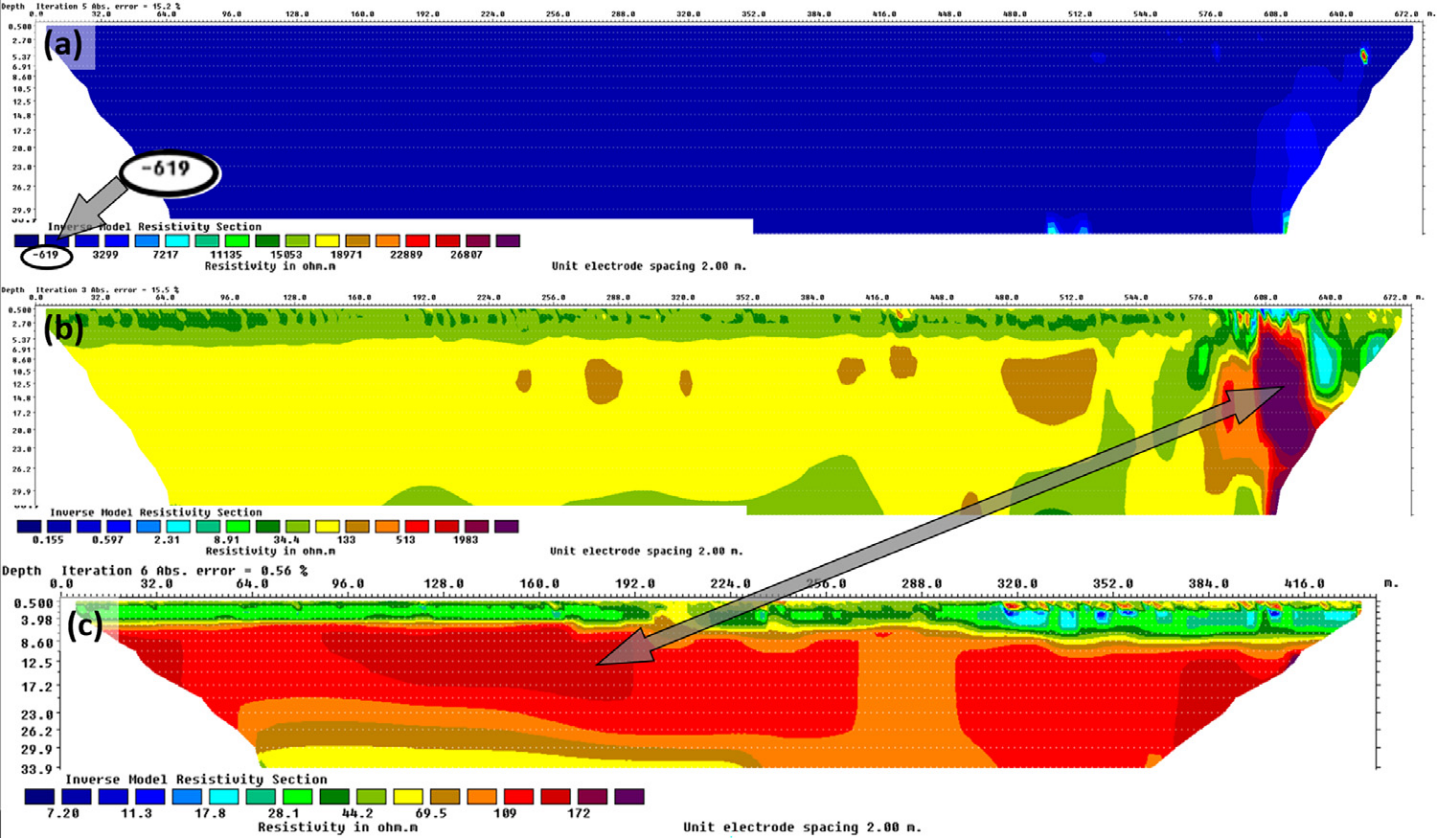
The inverted resistivity models: (a) ERI profile B (rain marred the end of measuring) – raw data; (b) ERI profile B (rain in the end of measuring) – postprocessing data; (c) ERI profile D results without rain (the arrow points at the equivalent fragments of the profiles).

While conducting the research campaign by the ERI profiling we faced problems with brief rain (water) impact on the current field; in such a case the Terrameter revealed false data with zero or negative resistivity values, or quite contrary, large resistivity variation (especially near surface). Such unexpected obstacles lead to the time-consuming results processing or even disabled the measurement. For example, during the ERI when conducting the line B measurements, by the end of profiling, the heavy rainfalls resulted in weird data values of the end part of the profiling section, and therefore, we could not analyse this section properly (Fig. 7a). We present the original *processed* (at a minimum) data, nevertheless, for better interpretation, it is recommended to use i.a. negative resistivity filters (an issue pointed out in the *Data description* chapter) and to try to visualize the data in customized contour intervals of the processing last iteration. We verified affected part of aforementioned section (Fig. 7b), (with poor quality in the end of profile) by overlapping next survey. Later, when the sky cleared up we measured the profile D of the same location as improper profile B – Fig. 7c shows that the profile D much differs in the problematic part from B section. A similar situation with the bad weather we experienced in the case of the ERI profile C.

## Ethics Statement

The authors declare no known ethical issues in respect of the data reported in this article.

## CRediT authorship contribution statement

**Łukasz Kaczmarek:** Conceptualization, Methodology, Investigation, Data curation, Validation, Writing – original draft, Writing – review & editing. **Grzegorz Sinicyn:** Conceptualization, Methodology, Investigation, Writing – review & editing, Funding acquisition. **Krzysztof Kochanek:** Investigation, Writing – review & editing. **Bartosz Bednarz:** Investigation, Writing – review & editing. **Mateusz Grygoruk:** Writing – review & editing, Funding acquisition. **Maria Grodzka-Łukaszewska:** Conceptualization, Methodology, Investigation, Writing – original draft.

## Data Availability

Electrical resistivity imaging data for hydrogeological and geological investigations of Szuszalewo peatland (North-East Poland) - original data (Original data) (Mendeley Data).
